# Model Systems of Motor Neuron Diseases As a Platform for Studying Pathogenic Mechanisms and Searching for Therapeutic Agents

**Published:** 2015

**Authors:** K. R. Valetdinova, S. P. Medvedev, S. M. Zakian

**Affiliations:** Institute of Cytology and Genetics, Prospekt Lavrentyeva, 10, Novosibirsk, 630090, Russia; Institute of Chemical Biology and Fundamental Medicine, Prospekt Lavrentyeva, 8, Novosibirsk, 630090, Russia; Meshalkin Novosibirsk State Research Institute of Circulation Pathology, Rechkunovskaya Str., 15, Novosibirsk, 630055, Russia; Novosibirsk State University, Pirogova Str., 2, Novosibirsk, 630090, Russia

**Keywords:** amyotrophic lateral sclerosis, induced pluripotent stem cells, motor neurons, spinal muscular atrophy, embryonic stem cells

## Abstract

Over the past 30 years, many molecular genetic mechanisms underlying motor neuron diseases (MNDs) have been discovered and studied. Among these diseases, amyotrophic lateral sclerosis (ALS), which causes the progressive degeneration and death of central and peripheral motor neurons, and spinal muscular atrophy (SMA), which is one of the inherited diseases that prevail among hereditary diseases in the pattern of child mortality, hold a special place. These diseases, like most nerve, neurodegenerative, and psychiatric diseases, cannot be treated appropriately at present. Artificial model systems, especially those that are based on the use of embryonic stem cells (ESCs) and induced pluripotent stem cells (iPSCs), are of paramount importance in searching for adequate therapeutic agents, as well as for a deep understanding of the MND pathogenesis. This review is mainly focused on the recent advance in the development of and research into cell and animal models of ALS and SMA. The main issues concerning the use of cellular technologies in biomedical applications are also described.

## INTRODUCTION

In the central nervous system (CNS), motor neuron bodies are located in the motor cortex (upper or central motor neurons), in the nuclei of the cranial nerves of the brainstem, and in the anterior horns of the gray matter of the spinal cord (lower or peripheral motor neurons). The processes of these neurons (axons), being a part of the conduction tracts (pyramidal and extrapyramidal tracts), anterior roots of the spinal cord, and peripheral nerves reach the skeletal muscles to form the neuromuscular junction on muscle fibers that are innervated by these cells.

Neurodegenerative diseases that affect primarily this group of nerve cells are called motor neuron diseases (MNDs). These diseases are usually characterized by muscle atrophy and palsy that result in the death of patients [1]. Degenerative processes associated with spinal muscular atrophy (SMA), progressive muscular atrophy, spinal and bulbar muscular atrophy (Kennedy’s disease), and hereditary motor neuropathies affect lower motor neurons and their processes [2]. Upper motor neurons are mainly affected by primary lateral sclerosis, hereditary spastic paraplegia, progressive bulbar and pseudobulbar palsy, and spinal muscular atrophy with respiratory distress type I [2, 3]. Both the central and peripheral motor neurons are involved in the pathological process associated with amyotrophic lateral sclerosis (ALS) [1].

Of greatest interest are SMA, which is the most common inherited neurodegenerative disease, particularly in children, and ALS, which is an extremely heterogeneous disease whose molecular mechanisms are understudied. The challenging issue is to develop adequate model ALS and SMA systems, since investigation of pathological processes in CNS cells caused by motor neuron diseases is currently impossible due to the lack of non-invasive and safe intravital techniques, while a postmortem examination of patient tissues provides insight only into the terminal stages of the disease. The problem can be solved in two ways.

The first path is to generate animal models that express the human genes involved in the pathogenesis of these diseases. However, such model systems, for obvious reasons, do not have all the genotypic and phenotypic features typical of human MND. Therefore, the second approach is an actively developed one that is based on the production of motor neurons derived from human pluripotent cells possessing a particular phenotype of ALS or SMA.

So-called pluripotent cells have the capability of differentiating into derivatives of all three primitive germ layers (entoderm, mesoderm, and ectoderm), cells of the inner cell mass (ICM), and the epiblast of mammalian embryos before [4] and after implantation [5], as well as embryonic germ cells. Cells derived from ICM and the epiblast of preimplantation embryos, which are cultured *in vitro *and preserve the properties of their precursors for a long time, were called embryonic stem cells (ESCs). The first human ESC lines were produced in 1998 [6].

In 2006, a group of Japanese scientists led by S. Yamanaka developed a method for reprogramming somatic cells to a pluripotent state by the expression of four factors: Oct3/4, Sox2, c-Myc, and Klf4 [7]. The characteristics of the resulting cells were close to those of ESCs, and, therefore, the cells were called induced pluripotent stem cells (iPSCs).

ESC- or iPSC-derived motor neurons serve as a platform not only for modeling diseases, but also for screening drugs and developing therapy techniques for MNDs and spinal cord injuries [8, 9]. They can be used in cell replacement therapy for affected nerve cells, as well as microenvironment components producing neurotrophic factors and processing toxic metabolites. The therapeutic effect of the transplantation of neural stem cells, which exert a paracrine effect on the immediate cell environment, was observed in several models of neurodegenerative diseases [10, 11]. To enhance this effect, production of certain neurotrophic factors *in vitro* can artificially be modulated. In this case, the transplanted cells will secrete recovery-associated factors into damaged tissue, as it was demonstrated in an ALS model in rats (Gly93Ala) transplanted with human neural progenitor cells expressing the glial-derived neurotrophic factor (GDNF) [12].

This review describes the main known model systems of ALS and SMA. Particular attention is focused on *in vitro *systems as well as on the application of cell technologies in practice.

## AMYOTROPHYC LATERAL SCLEROSIS

**General characteristics**

Amyotrophic lateral sclerosis (ALS) (also known as Lou Gehrig’s disease) was first described in detail by the prominent French doctor, a specialist in the field of neurological diseases, Jean-Martin Charcot in 1869. The very name reflects the distinctive features of the disease: muscle atrophy (amyotrophic) due to selective injury to peripheral motor neurons of the anterior horns of the spinal cord and the brainstem motor nuclei, as well as cortical motor neurons and the lateral columns of the spinal cord (lateral sclerosis) [13]. Patient death usually occurs due to complete failure of the respiratory muscles 2–5 years after the onset of the first symptoms [14].

ALS is an orphan disease whose rate in different populations ranges from one-two to four-six cases per 100,000 people per year [15-17]. Currently, about 25,000 patients with a mean age of 55 years are listed in the U.S. for ALS. In addition, ALS occurs in males more often than in females (3 : 2 ratio) [18].

Sporadic and familial (or inherited) forms of ALS can be distinguished, with the fraction of the sporadic form accounting for about 90% of all cases of the disease. The risk factors for ALS include the influence of heavy metals and toxins (e.g., the natural cyanobacteria toxin β-N-methylamino-*L*-alanine), smoking, severe traumatic brain injuries, increased motor activity, latent viral and non-viral infections, and autoimmune reactions [19-26].

According to modern concepts, the inherited form of ALS is linked to mutations in 12 genes [1]. In total, the development of ALS is associated with mutations in 116 genes, which are presented in the constantly updated Amyotrophic Lateral Sclerosis Online Database (ALSoD) [27]. These are mainly single nucleotide substitutions in the coding region of genes, deletions, insertions, and expansion of repetitive sequences. The most common genetic causes of ALS include expansion of the GGGGCC hexanucleotide repeats in the first intron/promoter of the *C9ORF72 *gene [28-30], as well as mutations in the genes *SOD1 *(*superoxide dismutase 1*, encodes Cu/Zn-binding superoxide dismutase 1) [31], *TDP-43 *(*TAR DNA-binding protein 43*) [32], *FUS *(*fused in sarcoma*, RNA-binding protein FUS) [33, 34], *ANG *(*angiogenin*, ribonuclease) [35],* OPTN *(*optineurin*) [36], and *VCP *(*valosin containing protein*) [37].

SOD1 is expressed in all cell types and localized in the cytoplasm. This protein catalyzes the conversion of the superoxide anion-radical into free oxygen and hydrogen peroxide. *SOD1 *gene mutations are the most numerous ones (more than 160) [1], but not all of them lead to the formation of a non-functional protein product that would explain the key role of oxidative stress and mitochondrial dysfunction in the ALS pathogenesis. TDP-43 and FUS are multifunctional proteins involved in gene expression and regulation of expression, including transcription, RNA processing, transport and translation, as well as miRNA synthesis. Cytoplasmic aggregates of TDP-43 and FUS are detected in patients with frontotemporal dementia (FTD) [38, 39]. The protein product of *ANG *gene is involved in transcriptional regulation. ALS-associated mutations of *OPTN *activate the transcription factor NF-κB and also affect the distribution of optineurin in the cytoplasm. VCP is involved in a variety of cellular processes, including the cell cycle regulation, formation of the nuclear envelope, and Golgi biogenesis. It is also a component of the ubiquitin- dependent proteolytic system [40].

ALS affects not just motor, but also other types of neurons, and some ALS forms are combined with FTD or degeneration of the dopaminergic neurons located in the midbrain structures in the basal ganglia (striatum), limbic system (hippocampus), and hypothalamus. Histological changes in several types of neurons, including cells of the hippocampus and basal ganglia, are detected even in patients whose clinical picture is dominated by dysfunction of the motor system [41].

However, despite numerous studies, there are still no methods of effective therapy for ALS, and treatment is actually limited to relieving the symptoms. For example, the drug riluzole, a glutamate-release inhibitor exhibiting neuroprotective properties, can modulate the course of ALS, increasing the lifespan of patients by 2–3 months, but without relieving the symptoms [42]. The NeuRx Diaphragm Pacing System is approved for use in the USA. This system enables to extend, for several months, the time during which ALS patients can breathe independently without mechanical ventilation.

The development of appropriate model ALS systems should help search for effective drugs and answer the question of how these diverse molecular changes lead to selective death of motor neurons.

**Main laboratory ALS models**

**Fig. 1:**
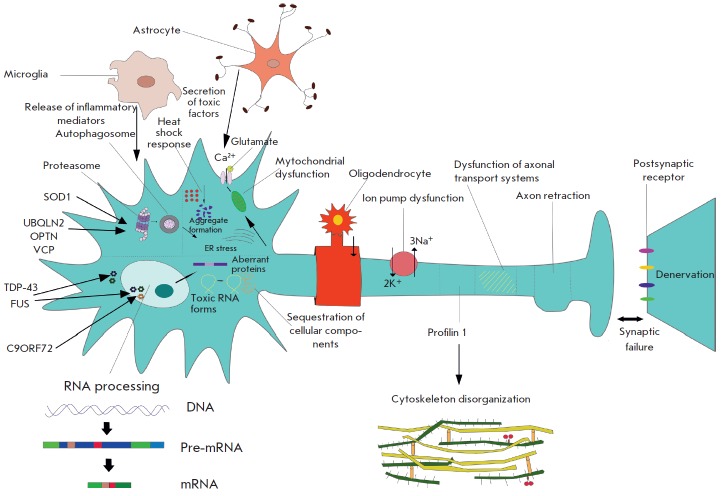
A general scheme of ALS etiopathogenesis. Mutations in *SOD1*, *VCP*, *UBQLN2*, *OPTN*, *CHMP2B, *and, possibly,* TARDBP *cause changes in protein degradation systems, disrupting the normal proteasomal and autophagic disposal. Mutations in *C9ORF72*, *TARDBP, *and *FUS *disturb RNA processing that leads to the formation of a large number of aberrant (incorrectly assembled) proteins and toxic RNA forms. These changes lead to intracellular proteinopathy that is characterized by the development of clusters and granules, endoplasmic reticulum and Golgi stress, and mitochondrial dysfunction. Disorganization of the axonal cytoskeleton and dysfunction of the axonal transport systems lead to denervation of motor neurons located downstream in the signal transmission chain (peripheral motor neurons), or muscle fibers. Cells that do not belong to neurons, including astrocytes, microglia, and oligodendrocytes, modify this process, because they cannot provide normal functioning of nerve cells and, in addition, possess a toxic effect. Factors determining the level of sensitivity to damages, including factors that modulate the type of stress response (activation of heat shock proteins) and provide “predisposition” to excitotoxicity (features of glutamate receptors) define exactly which neurons will be affected by these processes to the greatest extent. The effect of proteins, such as profilin 1 and the neurofilament heavy chain (NFH), on this model is revealed at a considerable distance from the nerve cell body. They directly affect the cytoskeleton and *D*-amino acid oxidase, which plays an important role in excitotoxicity. The systems involved in the signaling processes of axonal “targeting” (e.g., semaphorin family proteins) as well as in determining the topography of connections in the nervous system (e.g., proteins of the ephrin and reticulon families) apparently trigger the processes of axon retraction and denervation

The generation of animal ALS model systems has made it possible to deepen our understanding of the disease and to identify a number of mechanisms leading to the development of ALS, including mitochondrial dysfunction, protein misfolding (wrong packaging) and protein aggregation, oxidative stress, glutamate excitotoxicity, non-cell-autonomous effects, inflammatory processes in the nervous tissue, axonal transport dysfunction, RNA processing dysfunction, etc (*Fig. 1*).

Mice carrying mutations in the *SOD1 *gene were generated in the early 1990s [31]. Mice and rats with various mutations in this gene are the most thoroughly studied animal model of ALS (*Table*). These animals have a lethal phenotype with a late onset. The phenotype is characterized by muscle denervation, activation of astrocytes and microglia, and loss of motor neurons in the spinal cord. This phenotype can be induced by overexpression of the mutant SOD1 protein; therefore, animals overexpressing the normal protein should serve as a control in these experiments.

**Table 1:** Animal models of amyotrophic lateral sclerosis

Model object	Gene	Phenotype	Reference
*Saccharomyces cerevisiae*	SOD1, TARDBP, FUS	Damage of mitochondrial membrane integrity, TDP-43 and FUS aggregation.	[155-158]
*Caenorhabditis elegans*	SOD1, TARDBP, FUS, tdp-1	Uncoordinated movements and locomotor impairments, palsy, degeneration of motor neurons, synaptic transmission failure, nuclear accumulation of TDP-43 aggregates, SOD1 aggregation.	[159-164]
*Drosophila melanogaster*	SOD1, TARDBP, FUS	Motor defects, stress activation of glial cells, SOD1 aggregation, gliosis, axonal degeneration, neuronal atrophy. In general, the effects vary depending on the tissue that expresses normal/mutant SOD1, TARDBP and FUS proteins.	[165-173]
*Danio rerio*	SOD1, TARDBP, FUS, Sod1	Motor defects, muscular atrophy, loss of motor neurons, reduced survival.	[174-176]
*Mus musculus * *Rattus norvegicus *	TARDBP, SOD1, Sod1, Tardbp	ALS phenotype: tremor, progressive motor disorders and palsy, gliosis, ubiquitinated SOD1 inclusions, degeneration of axons and motor neurons, vacuolization of mitochondria, rare cytoplasmic aggregates of phosphorylated TDP-43.	[48, 51, 177-192]
Dog breeds: Pembroke Welsh corgi, Boxer, Rhodesian ridgeback, German Shepherd, Chesapeake Bay	SOD1	Degenerative myelopathy of dogs: inclusions capable of binding with SOD1 antibodies are observed in the cytoplasm of neurons; demyelination of the white matter of lateral cords and axonal loss.	[193, 194]
*Macaca fascicularis*	TDP-43	Accumulation of TDP-43 aggregates and cystatin C-positive granules in the cytoplasm; progressive motor weakness of the distal portions of the upper extremities, fasciculations and atrophy.	[52]

The effects of TDP-43 insufficiency have been studied in different model organisms (*Table*). TDP-43 knockout in *Drosophila melanogaster *leads to a variety of neuromuscular defects [43], and TDP-43 knockdown in zebrafish (*Danio rerio*) causes decreased motor axons length and branching [44]. In mice, homozygous deletion of the *Tardbp *gene, which encodes TDP-43, is lethal, but only moderate motor defects are observed in heterozygous animals [45]. Overexpression of mutant TDP-43 in yeast, nematodes, and *D. rerio *induces more serious disturbances compared to normal protein overexpression [44-46]. An elevated expression of the normal or mutant TDP-43 protein in rodents led to the formation of a phenotype with cortical disorders with the involvement, in a number of cases, of peripheral motor neurons [47-51]. Overexpression of TDP-43 in the spinal cord of the cynomolgus monkey (*Macaca fascicularis*) induced a progressive loss of motor neurons [52].

Some deletions in the *Fus *gene in mice were demonstrated to be lethal or to induce a phenotype not associated with neurodegeneration [53, 54]. Mice with FUS knockout in hippocampal neurons have a reduced amount of dendrites and pronounced morphological defects of these processes [55]. Overexpression of the normal human FUS protein in transgenic mice caused active degeneration of motor neurons that was characterized by the formation of globular and “skein-like” FUS-positive inclusions in the motor neurons [56]. In rats, overexpression of FUS with an Arg521Cys mutation led to the death of cortical, hippocampal, and motor neurons, as well as to denervation and development of palsies [57].

Therefore, these ALS models demonstrate the important role of the proteins SOD1, TDP-43, and FUS in the functioning of different cells of the nervous system, including motor neurons.

**ALS cell models**

To date, cell models of both the hereditary and sporadic forms of ALS have been generated (*Table 2*). However, technologies and approaches that use a patient’s iPSCs are mainly utilized not for a direct searching for therapy approaches, but for the identification and profound analysis of the pathogenic mechanisms of this neurodegenerative disease.

**Table 2:** Cell models of amyotrophic lateral sclerosis

Gene	Mutation	Phenotype	Reference
TDP-43	Met337Val Gln343Arg Gly298Ser	Reduced survival, increased sensitivity to PI3K kinase inhibition, elevated level of the TDP-43 protein.	[65, 68-70]
SOD1	Gly85Ser Leu144Phe Ala4Val Asp90Ala Asn87Ser Ser106Leu	Hyperexcitability of membranes, neurofilament aggregation, mitochondrial dysfunction, oxidative stress and endoplasmic reticulum stress.	[58, 60, 146, 195, 196]
FUS	His517Gln	Hyperexcitability of membranes, FUS aggregates.	[60]
C9ORF72	Expansion of the GGGGCC hexanucleotide repeat in the first intron/promoter.	Abnormal electrophysiologic indicators, hyperexcitability of membranes, formation of focal granules of C9ORF72 RNA containing hnRNPA1 and Pur-α proteins.	[60, 71]
Sporadic form		Intranuclear aggregates of the hyperphosphorylated TDP-43 protein.	[75]

**Cell models of the inherited ALS form**

***SOD1.*** Motor neurons containing the *SOD1 *gene with an Asp90Ala mutation demonstrate signs of neurofilament aggregation that lead to the degeneration of neurites [58]. The mutant SOD1 protein was found to be capable of binding to the 3’-untranslated region of mRNA of one of the neurofilament components, NF-L, decreasing the mRNA stability. Thereby, the proportion of individual subunits of neurofilaments in motor neurons is disturbed. This is the interaction that can trigger a chain of events that lead to selective death of motor neurons [58].

Defects in the mitochondrial transport system and changes in the mitochondrial morphology have been found in motor neurons with an Ala4Val missense mutation in the *SOD1 *gene. Manifestations of oxidative stress and endoplasmic reticulum stress, as well as activation of the unfolded protein response (UPR), were observed in these cells [59]. Furthermore, an analysis of high-throughput mRNA sequencing using the DAVID and GSEA platforms demonstrated that gene transcription in motor neurons with the *SOD1^+/A4V^*genotype is altered compared to the isogenic control without this mutation. Motor neurons with a *SOD1 *mutation had an increased transcription level of genes encoding contractile proteins, in particular kinesins, as well as the genes involved in cytoskeleton formation and transcription regulation. In this case, the transcription level of the genes involved in the functioning of mitochondria and translation was significantly decreased in these cells [59].

An electrophysiological study of iPSC-derived motor neurons with mutations in the *SOD1 *gene, as well as in* C9ORF72 *and *FUS, *revealed the hyperexcitability of their membranes that may be the main element of the ALS pathogenesis, leading to the death of motor neurons [60]. A decrease in the amplitude of the delayedrectifier potassium current was observed in these cells, which might be the cause of the hyperexcitability of their membranes. The use of a potassium channel activator, retigabine, blocked the hyperexcitability and increased the degree of survival of motor neurons with mutations in the *SOD1 *gene [60].

Screening of mouse ESCs with mutations in *SOD1* revealed a number of potential drugs [61]. A relationship between glycogen synthase kinase 3 (GSK-3) and ALS was previously identified [62]. Inhibition of the GSK-3 pathway was found to reduce neuronal apoptosis [63, 64]. One of the inhibitors of this pathway, kenpaullone, caused a significant increase in the viability of mouse motor neurons with mutations in *SOD1, *and it also increased the degree of survival of the motor neurons produced after differentiation of thee iPSCs of ALS patients [61].

In addition, the primary culture of mouse glial cells expressing a mutant (Gly93Ala) human SOD1 protein exerts an increased toxic effect on motor neurons. Most likely, the ALS pathogenesis occurs through a non-autonomous mechanism in the case of mutations in *SOD1* [65, 66].

***TDP-43.*** TDP-43 protein aggregates in motor neurons are detected in 97% of ALS cases and in 45% of FTD cases [67]. Motor neurons derived from iPSCs with a Met337Val missense mutation in the *TDP-43 *gene were found to have an increased level of the soluble and detergent- resistant TDP-43 protein, reduced survival in long-term cultivation, and also increased sensitivity to PI3K kinase inhibition [68].

Investigation of astrocytes produced from mutant iPSCs (Met337Val) demonstrated an increased level of the TDP-43 protein in astrocytes, same as in motor neurons, with protein aggregates being mainly found in the cytoplasm of the cells. These cells also showed reduced survival in the culture [65]. The co-culture of mutant astrocytes with control and mutant motor neurons demonstrated that the presence of astrocytes does not affect the viability of motor neurons. This indicates that the ALS pathogenesis occurs via the cell-autonomous pathway in the case of mutations in *TDP-43 *[65].

Motor neurons differentiated from patient iPSCs carrying Met337Val, Gln343Arg, and Gly298Ser mutations in *TDP-43 *had an increased amount of the insoluble TDP-43 protein bound to the SNRPB2 spliceosomal protein [69]. Furthermore, these cells had an increased transcriptional level of the genes involved in the RNA metabolism and a reduced transcriptional level of the genes encoding cytoskeleton proteins. Four compounds that are inhibitors of the enzymes involved in covalent modification of chromatin and the proteins associated with RNA splicing were tested: trichostatin A (histone deacetyltransferase inhibitor), spliceostatin A (inhibitor of spliceosomal proteins), anacardic acid, and garcinol (histone acetyltransferase inhibitors). Anacardic acid appeared to be capable of increasing the chance of survival of mutant motor neurons, decreasing the transcriptional level of the *TDP-43 *gene mRNA and the TDP-43 protein level in the insoluble fraction, as well as increasing the length of motor neuron neurites [69].

iPSCs can be used not only to search for new compounds as potential drugs for ALS, but also to explore alternative modes of therapy; e.g., via RNA interference. Based on the design of small interfering RNAs (siRNA) designated for allele-specific suppression of the translation of a mutant (Met337Val) TDP-43 protein [70], the use of siRNA was demonstrated to be capable of a 30% reduction in the cytoplasmic TDP-43 protein level in neural stem cells derived from patient iPSCs [70].

***C9ORF72.*** RNA of the mutant *C9ORF72 *gene with an abnormal number of GGGGCC hexanucleotides in the first intron/promoter can also initiate a pathological process associated with ALS. An increased transcriptional level of *C9ORF72, *as well as the formation of focal accumulations of *C9ORF72 *RNA, containing, among other things, hnRNPA1 and Pur-α RNA-binding proteins, was observed in motor neurons produced after the differentiation of iPSCs from patients with the C9-ALS familial form (hexanucleotide repeat expansion in the *C9ORF72 *gene) [71]. hnRNPA1 is known to bind to TDP-43 molecules [72]. Therefore, the interaction of TDP-43 with its target RNAs probably changes upon removal of hnRNPA1 from focal accumulations. Therefore, there is a potential relationship between two ALS forms (C9-ALS and TDP-43-mediated ALS). Furthermore, mutations in the hnRNPA1 and hnRNPA2/ B1 proteins were found to be one of the causes of MND in humans [73]. What is more, Pur-α was shown to interact with focal accumulations of RNAs containing the GGGGCC repeats and to modulate the toxic effect of similar structures in an ALS model in* D. melanogaster *[74]. Cells expressing mutant RNA of the *C9ORF72 *gene had an altered expression level of the genes associated with the membrane excitability, in particular *DPP6*, and had abnormal electrophysiological indicators. The use of antisense oligonucleotides complementary to RNA of the *C9ORF72 *gene enabled the suppression of the formation of focal accumulations and recovery of the normal level of gene transcription in motor neurons [71]. These studies exemplify the fact that differentiated derivatives of iPSCs can be used to search for and explore potential drugs [61, 69].

**Cell models of the sporadic ALS form**

Using patients with the sporadic ALS form, Burkhardt* et al*. [75] produced iPSC lines with a unique genetic and epigenetic background. The formation of hyperphosphorylated aggregates of the TDP-43 protein was observed in the nuclei of motor neurons differentiated from these cells after 2 months of cultivation [75], but no accumulation of ubiquitin-labeled TDP-43 granules was found. This suggests that TDP-43 is exposed to ubiquitination at the later stages of proteinopathy compared to hyperphosphorylation. The authors note that it is important to differentiate iPSCs derived from different patients not only into motor neurons, but also into other cell types in order to investigate the causes behind the wide variety of sporadic ALS cases. This model is of particular interest for the search for therapeutic agents and factors that modify ALS.

## SPINAL MUSCULAR ATROPHY

**General characteristics**

Spinal muscular atrophy (SMA) is a neurodegenerative disorder with an autosomal recessive mode of inheritance that is characterized by degeneration of motor neurons in the anterior horns of the spinal cord that leads to muscle atrophy, palsy, and death of the patient [76-78]. Spinal muscular atrophy in children was first described by G. Werdnig in 1891. The disease’s frequency in European populations is 1 per 10,000 newborns, and the carrier frequency of the mutant gene is 1 per 40–50 [79].

**Fig. 2:**
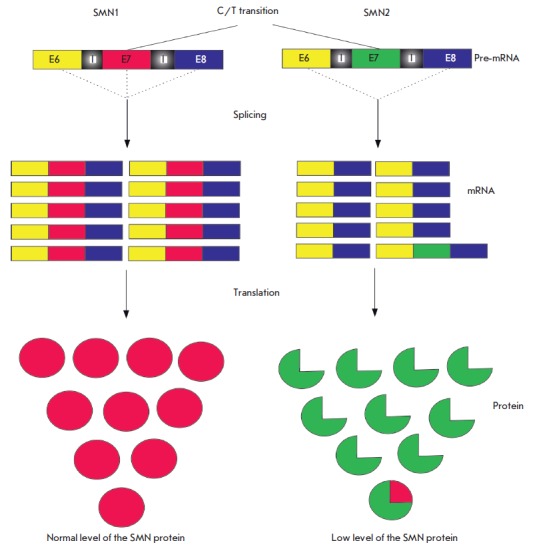
Expression of the *SMN1 *and *SMN2 *genes (see the text for the description)

Over 95% of SMA patients have a homozygous deletion in the *SMN1 *(*Survival Motor Neuron1*) gene located on chromosome 5, while inversions, reading frame shift mutations, missense mutations, nonsense mutations, and splicing site changes occur only in a few cases [80, 81]. A full list of known mutations of the *SMN1* gene is available in the Leiden Open Variation Database (http://www.dmd.nl/nmdb2/home.php?select_ db=SMN). The *SMN2 *pseudogene, which differs from* SMN1 *only in eight single nucleotide substitutions by one in the seventh and eighth exons, and the other substitutions occurring in introns, is located on the same chromosome [82]. A C/T transition in exon 7 leads to a change in the splicing of the *SMN2 *transcript, such that 90% of translated RNAs do not contain exon 7, and the protein product is unstable and shortened [83, 84] (*Fig. 2*). In this case, the number of pseudogene copies in the genome of different individuals can vary from 0 to 6. The larger the number of SMN2 copies, the lesser the severity of SMA symptoms [85-87]. The *SMN2* gene significance for the development of a more mild form of spinal muscular atrophy is confirmed by asymptomatic cases when the number of *SMN2 *gene copies is sufficiently large (four or more) in individuals homozygous for deletion of the *SMN1 *gene [88].

Over 95% of SMA patients have a homozygous deletion in the *SMN1 *(*Survival Motor Neuron1*) gene located on chromosome 5, while inversions, reading frame shift mutations, missense mutations, nonsense mutations, and splicing site changes occur only in a few cases [80, 81]. A full list of known mutations of the *SMN1* gene is available in the Leiden Open Variation Database (http://www.dmd.nl/nmdb2/home.php?select_ db=SMN). The *SMN2 *pseudogene, which differs from* SMN1 *only in eight single nucleotide substitutions by one in the seventh and eighth exons, and the other substitutions occurring in introns, is located on the same chromosome [82]. A C/T transition in exon 7 leads to a change in the splicing of the *SMN2 *transcript, such that 90% of translated RNAs do not contain exon 7, and the protein product is unstable and shortened [83, 84] (*Fig. 2*). In this case, the number of pseudogene copies in the genome of different individuals can vary from 0 to 6. The larger the number of SMN2 copies, the lesser the severity of SMA symptoms [85-87]. The *SMN2* gene significance for the development of a more mild form of spinal muscular atrophy is confirmed by asymptomatic cases when the number of *SMN2 *gene copies is sufficiently large (four or more) in individuals homozygous for deletion of the *SMN1 *gene [88].

Depending on the age of onset, severity, and lifespan, the following disease types are distinguished [89]:

Type I (Werdnig-Hoffmann disease) is the most severe form that manifests itself during the first 6 months of life and is characterized by pronounced signs of palsy of the limb and trunk muscles, as well as the respiratory muscles; children are unable to sit and to keep their head independently. The lifespan for this disease form does not exceed 2 years.

Type II is an intermediate form that has a later onset, usually at the age of 7–18 months. Sick children are capable of sitting independently but do not achieve the ability to walk. The lifespan is more than 2 years.

Type III (Kugelberg-Welander disease) is a mild/ moderate form. The first symptoms emerge after 18 months. Patients are able to achieve independent standing and walking.

Type IV is an adult form. In most cases, it starts after 20–30 years and does not significantly affect the lifespan. It manifests itself in weakness of the proximal muscles, fasciculations (involuntary, chaotic contractions of individual groups of muscle fibers), as well as reduced tendon reflexes.

A *SMN1 *gene protein product performs several functions in the cell: it is involved in pre-mRNA splicing, mature mRNA transport, and axonal growth [90-94]. SMN is a central component of the complex required for assembly of spliceosomal small nuclear ribonucleic particles (snRNPs) [95]. An association of spliceosomal components with each other in every splicing cycle is known to occur *ex novo *each time through stepwise assembly, which means that mutant SMN cannot provide effective assembly of snRNPs. Therefore, one of the hypotheses used to explain the SMA mechanism is based on the assumption that impaired snRNP formation affects the splicing of a specific group of genes that are important for the functioning of a motor neuron chain [95-97].

An axonal isoform of a protein product of the *SMN1* gene (a-SMN) was discovered in 2006 [98]. The axonal SMN transcript differs from the full-length transcript by the inclusion of the intron 3 sequence, but the protein translated from this transcript is shorter than the SMN protein because of the stop codon located on the boundary of exon 3 and intron 3. Therefore, the SMN and a-SMN proteins have an identical N-terminal region and a different C-terminal region. The a-SMNprotein was found to be selectively expressed in the critical phase of motoneuron development and to be localized mainly in axons, stimulating axonogenesis. Expression of this protein is reduced in adults [98]. However, the existence of the specific neuronal a-SMN isoform does not explain the important fact of a lacking exon 7 in the *SMN2 *gene mRNA in most SMA cases, because only the first four exons in a-SMN are encoding ones [99]. Therefore, the second hypothesis suggests that SMA is associated with impairment of the important function that is performed by SMN in the axons of motor neurons [91, 94-97, 99, 100]. Therefore, what is the cause of the selective death of a motor neuron in the presence of *SMN1 *mutations? And how can we help SMA patients? Artificial model systems should help answer these questions.

**Main animal SMA models**

The SMN protein deficiency has been studied in several model organisms (*Table 3*). However, working with animals is complicated by the fact that their genomes contain only one *Smn *gene that is equivalent to the human* SMN1 *gene, and they do not have the *SMN2 *gene. For this reason all *Smn *knockout animals die, and the time of death is determined by the SMN1 mRNA level inherited by a new organism from the mother. For example, death in mice occurs at the early stages of development [101], and death in egg-laying organisms, e.g. in *D. melanogaster*, occurs later, when the SMN protein level inherited from the mother decreases to a critical point [102]. As expected, *Smn *knockout in a specific tissue leads to the maldevelopment of this tissue and loss of a larger portion of its cellular component [103-105]. Additional copies of *SMN2 *are usually inserted into the genome of transgenic mice with SMA. Two copies of this gene ensure a greater chance of survival of embryos, while eight copies result in mice with a normal phenotype [106, 107]. Two *SMN2 *copies were shown to be sufficient for the normal functioning of most tissues; however, motor neurons require a higher SMN level, at least in mice [108].

**Table 3:** Animal models of spinal muscular atrophy

Object	Manipulations with the SMN (Smn) gene	Phenotype	Reference
*Schizosaccharomyces* *pombe*	Knockout	Death	[197-199]
*Caenorhabditis* *elegans*	Knockout, knockdown, point mutations.	Embryonic death, developmental defects, motor defects, decreased life span.	[109, 200, 201]
*Drosophila* *melanogaster*	Point mutations equivalent to silent alleles, mutations disorganized Smn protein in adult flies, knockdown.	Embryonic death, loss of the ability to fly and jump.	[102, 112, 202]
*Danio rerio*	Knockdown	Death, defects of axon development.	[91]
*Mus musculus*	Knockout, directed alteration of expression in specific tissues at a specific period of time, introduction of transgenes of the human SMN1 gene with known missense mutations, introduction of additional copies of SMN2.	Embryonic death, apoptosis of a cellular component of the tissue that does not express Smn, a phenotype varies depending on the mutation type and the presence of additional transgenes, two copies increase the life span of embryos up to 5 days.	[101, 103-107, 203]

To conduct laborious experiments, invertebrates and vertebrates are usually used that do not belong to the class of mammals. For example, full-scale molecular genetic screening of chemical agents, potential drugs, is easier to conduct in *C. elegans *and *D. melanogaster*. So, a nematode with a *smn-1(cb131) *mutation was used for selection of three substances that most effectively alter a mutant phenotype: 4-AP (potassium channel blocker), gaboxadol hydrochloride (GABAA receptor agonist), and Neu5Ac monosaccharide [109]. Therefore, this model can serve as a basis for the screening of compounds that modify the functions of the Smn protein.

The influence of the most effective substances is further studied in more complex objects: in particular in* D. rerio *and mice. There are data indicating that the RhoA GTP-ase and its effector, Rho-kinase (ROCK), involved in cytoskeleton formation are of great importance upon diseases of motor neurons. Introduction of ROCK inhibitors into mice with SMA increased their lifespan and improved the state of their neuromuscular synapses and skeletal muscle fibers [110]. These findings have been confirmed in humans. For example, a genome-wide methylation analysis revealed significant differences in the DNA methylation level of two genes,* CHML *and *ARHGAP22*, in SMA patients and healthy individuals. The products of these genes regulate the function of the Rho and Rab GTP-ases that are regulators of actin dynamics, and, therefore, they can affect initiation, growth, direction, and branching of axons [111].

The results obtained in various animal SMA models should be interpreted with caution. For example, survival of the SMN-deficient flies *D. melanogaster *can be achieved by expression of this protein in the muscle tissue [102, 112]. But, there is no such effect in SMA mice with expression of SMN in muscles [108]. However, it can be noted that SMN in these experiments was expressed in the mesodermal progenitors of muscle fibers in *D. melanogaster *and in already formed muscle fibers, which no longer divided, in mice.

**Cell SMA models**

To date, iPSCs of type I SMA patients have been produced [113-115]. These cells differentiate into motor neurons *in vitro *with the same initial efficiency as control cells without mutations of *SMN1 *in the genome [113, 114]. However, the number and size of motor neurons derived from SMA patients is significantly reduced during prolonged cultivation compared to those of motor neuron cultures from healthy donors [113]. This reduction is caused by an elevated level of apoptosis, mediated by the Fas-ligand, and activation of caspase-8 and caspase-3. In this case, the addition of antibodies specific to the Fas-ligand and use of a caspase-3 inhibitor decrease the level of motoneuron apoptosis [114].

In neurons and astrocytes, the SMN protein is located in the cytoplasm, while in the nucleus of nerve cells it is located in special structures, gems (gemini of coiled (Cajal) bodies), so named because of the similarity of their structure, functions, and proximity. The Cajal bodies, similar to the gems associated with them, are involved in the maturation, assembly, and transport of snRNAs [116]. The amount of gems in the nucleus was demonstrated to correlate with the SMA form [117]. The number of gems in healthy people corresponded to the number of Cajal bodies and they were easily detected. Only Cajal bodies, and no gems, were found in type I SMA patients, whereas gems were detected only in some nuclei in type III SMA patients [118, 119]. There were no gems in the nuclei of the neurons and astrocytes derived from the iPSCs of SMA patients. Addition of valproic acid and tobramycin, which are used in SMA therapy, significantly increased the number of gems in cell nuclei and the SMN protein level. However, both the total level of the SMN protein and the number of gems still remained significantly lower than those in cells from healthy donors [113].

In a study by Corti *et al*., iPSCs were obtained from SMA patients using nonviral, nonintegrated episomal vectors [115]. Then, the resulting cells were transfected with short single-stranded oligonucleotides complementary to 75 nucleotides of the coding strand of the gene. The central part of these oligonucleotides contained a substitution (the same as in exon 7 that prevents full protein formation). After recombination with this donor molecule, the *SMN2 *gene in some cells became the “SMN1-like gene”; i.e. it was translated to the normal full-length SMN protein. Motor neurons derived from these cells with the corrected phenotype were transplanted into the spinal cord of mice with SMA. As a result, some changes in the pathological phenotype, as well as an increased lifespan of sick mice, were observed. However, the positive dynamics was apparently due to the production of neurotrophic factors by the transplanted cells [115].

SMA-associated pathological changes are known to occur also in other cell types, including astrocytes, sensory neurons, Schwann cells, and skeletal muscle fibers [120-124]. Do sensory neurons with a mutation in the *SMN1 *gene affect the progressive degeneration of motor neurons? The use of iPSCs from type I SMA patients helps answer this question.

iPSC lines with the SMA genotype were differentiated into sensory neurons. In this case, a decrease in the calcium response to depolarizing stimuli was observed, but the survival of these cells did not differ from that of the control group cells [125]. The co-culture of sensory neurons from SMA patients and motor neurons from healthy donors revealed no significant reduction in the number of motor neurons, as well as the formation of clusters of glutamate transport vesicles near the bodies of the motor neurons and neurites. Therefore, in this system, sensory neurons carrying a mutation in *SMN1* was demonstrated not to contribute significantly to the death of motor neurons with the normal *SMN1 *gene.

**The use of modern methods of genomic engineering to generate artificial model systems**

Modern methods of genome editing that are based on the technologies ZFN (Zinc-Finger Nuclease), TALEN (Transcription Activator-Like Effector Nucleases), and CRISPR/Cas9 (Clustered Regularly Interspaced Short Palindromic Repeats/Cas9) enable one to produce artificial model systems both *in vitro *and *in vivo*. They can be used not only to introduce a certain mutation in the genome of the study subject, but also to repair mutations [126-134].

At present, the TALEN and CRISPR/Cas9 technologies can be used in basic and translational biomedical research and experiments to test hypotheses and principles of gene and cell therapy. Artificial nucleases can be used, apart from the generation of models for developing approaches to treatment, directly for therapeutic purposes. One such area is treatment of chronic viral infections [135-138].

It became possible to correct a mutation of the Ala- 4Val substitution in the *SOD1 *gene in iPSCs using a pair of ZFNs [59]. In this case, homozygous and heterozygous cell clones (SOD1^+/A4V^ and SOD1^+/+^) were generated. These cells were used to further investigate the functions of the mutant SOD1 protein and for the purpose of isogenic control.

**Cell therapy of MND**

Cell therapy for neurodegenerative diseases involves the replacement of the affected nervous tissue with new healthy cells and recovery of the disrupted functions. For example, motor neurons derived from human ESCs were transplanted into chick embryos, where they survived and retained their cell specificity. Furthermore, their axons extended beyond CNS and reached their peripheral muscle targets [139]. Similar cells transplanted into the spinal cord of adult rats also survived in a foreign tissue. A number of cells expressing a marker of motoneurons, choline acetyltransferase, were found 6 months after the operation. A stronger effect can be achieved by co-transplantation of neural stem cells secreting a glial-derived neurotrophic factor into the affected area and additional administration of a phosphodiesterase- 4 inhibitor and dibutyryl cyclic adenosine monophosphate, substances that stimulate peripheral axonal outgrowth, to these animals [140]. Transplantation of motor neurons into the distal ends of the peripheral nerves in mice stimulated the formation of neuromuscular synapses [141-143]. In this case, the formation of functional synapses that persisted for 6–18 months after the surgery was observed. And additional electrical stimulation of the surviving cells resulted in re-innervation of the atrophied muscle fibers [143].

Surgery for motor neuron transplantation is still associated with technical difficulties and immunological responses. However, transplantation of differentiated iPSC derivatives avoids the problems of tissue incompatibility observed upon using ESC derivatives. In addition, issues of co-transplantation of microenvironment cells, formation of peripheral functional neuromuscular synapses, and increase in the survival and time of the transplanted cell activity require further research.

**The problem of directed differentiation of motor neurons and experiment scaling in pharmacological studies**

**Fig. 3:**
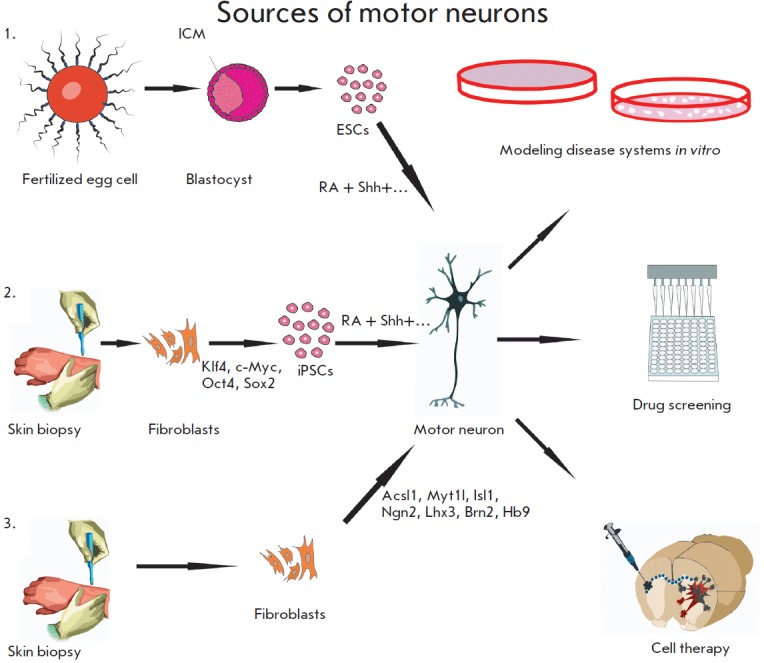
Sources of motor neurons. *1 *– ESCs derived from the inner cell mass of a blastocyst can be differentiated into motor neurons. Compounds like RA and Shh play a key role in this process. *2 *– human fibroblasts obtained from a skin biopsy material can be reprogrammed into iPSCs by expression of factors such as Klf-4, c-Myc, Oct4, and Sox2. iPSCs differentiation into motor neurons is performed by the method described for ESCs. *3 *– motor neurons can directly be produced from fibroblasts by expressing seven factors (Acsl1, Mt1l, Isl1, Ngn2, Lhx3, Brn2, Hb9)

Currently, motor neurons can be produced using three sources (*Fig. 3*): • ESCs; • iPSCs; and • fibroblasts. The development of protocols for fast and efficient differentiation of ESCs and iPSCs is extremely important, because differentiated derivatives of these cells are required for large-scale use in pharmacological and toxicological studies and cell replacement therapy. Currently, there is a large number of protocols for directed differentiation of cultured pluripotent human and mouse cells into motor neurons [71, 75, 115, 144-153]. This procedure includes two stages. The first stage is neuronal differentiation with the formation of embryoid bodies or neural rosettes. This stage is carried out in a ESC medium supplemented with specific factors that guide the differentiation towards neurons. The second step is differentiation of the resulting neural progenitors towards motor neurons by means of addition in the medium of factors such as RA (retinoic acid) and Shh (sonic hedgehog). The procedure efficiency is evaluated based on the expression of specific markers, morphology of the cells, their electrophysiological activity, as well as by xenotransplantation to animals. The resulting cells are a mixed population. It can be enriched with motor neurons by using gradient centrifugation [115] or protocols with a higher yield of the desired cells.

Protocols that use induction of the embryoid bodies followed by treatment with RA/Shh are quite laborious; they take a total of about 2 months, with a relatively low yield of motor neurons (10–40%). The method of directed programming that is based on adenoviral delivery of three motoneuron-specific transcription factors (Ngn2, ISL1, and Lhx3) is faster (formation of motor neurons from neural progenitors takes 11 days) and more efficient (motoneuron population amounts to about 60%). The disadvantages of this method are as follows:

• manipulations, which are based on the use of adenoviruses, with genomes that are relatively unsafe for further use of these cells; and

• considerable variations in the amount of produced motoneurons, as well as the variability of their survival.

However, protocols have already been developed for a fairly quick (within 20 days) and highly efficient (over 70%) production of motor neurons without the use of adenoviruses [154].

Further efforts should be aimed not only at searching for new, more effective methods of differentiation, ; but also at standardizing the parameters of cell passaging and culturing according to existing methods, as well as at studying procedures of direct differentiation of cells into specific motor neuron subtypes.

**The problem of generating cell model biobanks**

The essential requirement in performing pharmacological and toxicological studies and cell therapy is the availability of cell samples obtained from patients with rare diseases. This gives rise to an urgent need for the generation of banks of human ESC and iPSC lines. This task requires employees with a high level of competence, the development of a specialized infrastructure, and strict quality control of samples. The world scientific community has long been concerned about this issue. The criteria to be met by banks of human ESC and iPSC lines are addressed in new programs such as CCRM (http://ccrm.ca/), CIRM (http://www.coriell.org/media-center/coriell-in-the-news/coriellawarded- 10mm-for-induced-pluripotent-stem-cellprogram), HiPSCi (http://www.hipsci.org), and Stem- BANCC (http://www.stembancc.org/).

One of the possible ways to achieve this important goal may be to use a crowdsourcing platform as, for example, is already the case in resources such as the Zebrafish Gene Collection, ADDGENE, PubMed, and the Drosophila “Red Book”. In the USA, there is already a prototype of a similar organization based on NIH (the National Institutes of Health, in particular NCATS (National Center for Advanced Translational Science) and NIHCRM (the NIH Center for Regenerative Medicine)). The collections of three organizations, RUCDR Infinite Biologics (Rutgers), the Coriell Institute for Medical Research (Coriell), and Wisconsin Stem Cell Bank (WISC), already include hundreds of ESC and iPSC lines received from various institutions.

Therefore, a number of issues need to be addressed in order to generate biobanks of cell models. The first issue is related to joining the efforts of the international community to ensure that researchers around the world can enjoy unfettered access to this biobank. The problem of biosafety and compliance of a biobank with the legislation of different countries is no less important. The second issue is the generation of a single database, where all the necessary characteristics of cell lines should be spelled out. The third issue is related to the rapid progress in the field of cell technologies. Less than 10 years after its creation, the iPSC technology has reached a level of development that already allows the use of these cells in preclinical trials of drugs, as well as their application in the field of regenerative and personalized medicine.

## CONCLUSIONS

The problem of neurodegenerative diseases and finding ways to treat them becomes the most urgent ones due to the increased lifespan in developed countries, since most of these diseases develop in old and senile age. Motor neuron diseases do not prevail in the overall pattern of mortality from neurodegenerative diseases, but they are the absolute leaders in the severity of progression and rate of death. Amyotrophic lateral sclerosis (ALS) causes progressive muscular atrophy and death due to respiratory failure within 2–5 years, and the most severe form of spinal muscular atrophy (SMA), the Werdnig-Hoffmann disease, leads to muscle atrophy, palsy, and death of sick children within the first 2 years of life.

MND modeling in *in vivo *systems using organisms, such as nematodes, drosophila, laboratory mice, and rats, has significantly expanded our understanding of the causes and mechanisms of MND pathogenesis and revealed a number of chemical compounds that could be used as treatment for these diseases. However, at the genotypic and phenotypic level, these models are very different from that which is observed upon MND in humans. Therefore, differentiated derivatives of ESCs and iPSCs are extensively used at present to develop relevant model systems. They can be used not only to study the disease features at the molecular, subcellular, and cellular levels, but also to exploit these cells in the future for replacement therapy and screening of new drugs. The highest prospects are associated with the possibility of transplantation of iPSC derivatives, because these cells are autologous to an intended donor that allows one to avoid immunological rejection reactions and promotes the development and implementation of a new phase of modern medicine, the era of personalized medicine.

The major problem that needs to be solved to reach this stage is the development of open-access banks of ESC and iPSC lines containing full information on each cell line. Today, the National Institutes of Health in the USA and a number of organizations in some developed countries are the most active ones in this area. However, combining the efforts of the world scientific community, including the scientific organizations and institutions of the Russian Federation, is required to create a more complete bank of ESC and iPSC lines.

## Abbreviations

MND: motor neuron disease
ALS: amyotrophic lateral sclerosis
FTD: frontotemporal dementia
iPSCs: induced pluripotent stem cells
SMA: spinal muscular atrophy
ESCs: embryonic stem cells
CNS: central nervous system
